# Quantum dots-based “chemical tongue” for the discrimination of short-length Aβ peptides

**DOI:** 10.1007/s00604-023-06115-0

**Published:** 2024-01-15

**Authors:** Klaudia Głowacz, Marcin Drozd, Weronika Tokarska, Nina E. Wezynfeld, Patrycja Ciosek-Skibińska

**Affiliations:** 1 Chair of Medical Biotechnology, Faculty of Chemistry, Warsaw University of Technology, Noakowskiego 3, 00-664 Warsaw, Poland; 2 Centre for Advanced Materials and Technologies CEZAMAT, Poleczki 19, 02-822 Warsaw, Poland

**Keywords:** Excitation-emission matrix fluorescence spectroscopy, 2D fluorescence, Multispectral fluorescence spectroscopy, Machine learning, Quantum dots, Chemical tongue, Peptide sequence recognition, Aβ peptides, Alzheimer’s disease, Short-length peptides

## Abstract

A “chemical tongue” is proposed based on thiomalic acid-capped quantum dots (QDs) with signal enrichment provided by excitation-emission matrix (EEM) fluorescence spectroscopy for the determination of close structural analogs—short-length amyloid β (Aβ) peptides related to Alzheimer’s disease. Excellent discrimination is obtained by principal component analysis (PCA) for seven derivatives: Aβ_1-16_, Aβ_4-16_, Aβ_4-9_, Aβ_5-16_, Aβ_5-12_, Aβ_5-9_, Aβ_12-16_. Detection of Aβ_4-16_, Aβ_4-16_, and Aβ_5-9_ in binary and ternary mixtures performed by QDs-based chemical tongue using partial least squares-discriminant analysis (PLS-DA) provided perfect 100% accuracy for the two studied peptides (Aβ_4-16_ and Aβ_4-16_), while for the third one (Aβ_5-9_) it was slightly lower (97.9%). Successful detection of Aβ_4-16_ at 1 pmol/mL (1.6 ng/mL) suggests that the detection limit of the proposed method for short-length Aβ peptides can span nanomolar concentrations. This result is highly promising for the development of simple and efficient methods for sequence recognition in short-length peptides and better understanding of mechanisms at the QD-analyte interface.

**Graphical Abstract:**

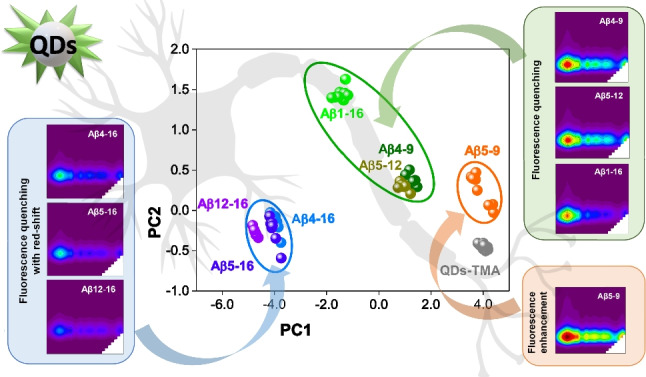

**Supplementary Information:**

The online version contains supplementary material available at 10.1007/s00604-023-06115-0.

## Introduction

The “chemical tongue” is an attractive sensing strategy to discriminate structurally similar compounds [1, 2]. As opposed to the traditionally applied detection using the lock and key method, its significant advantage is no need for designing specific receptor elements because the detection is realized using multiple cross-reactive receptors or multimodal response of single receptors. The resulting chemical “fingerprints” are then processed using machine learning algorithms to decode useful knowledge from the signal patterns, i.e., to obtain qualitative and/or quantitative information [1, 3]. Among many receptors used in “chemical tongues,” different nanomaterials are distinguished by favorable physicochemical properties, including tunable optical properties (e.g., plasmonic, photoluminescent) combined with the ease of surface functionalization [4]. Moreover, specially designed nanomaterials may supply multidimensional optical information, which can be beneficial for the analysis’s overall time- and cost-effectiveness in chemical tongue approach [5]. The idea of capturing multidimensional optical information may be realized using advanced detection techniques such as excitation-emission matrix (EEM) fluorescence spectroscopy, covering the entire emission spectrum at multiple excitation wavelengths [6]. As we showed in our recent work, the resultant EEM spectrum encodes subtle differences in the impact of the studied neurotransmitters on the fluorescent properties of QDs, which in turn improves the efficiency of the analytes identification, compared to the traditional analysis based on single emission spectrum [7].

Amyloid β (Aβ) peptides are a family of peptides generated from an amyloid precursor protein (APP). The longer Aβ forms tend to aggregate due to the hydrophobic character of the C-terminal fragment, whereas their N-terminal part could bind metal ions, including copper ions, forming highly redox active species. Both processes are believed to be related to Alzheimer’s disease (AD), leading to the formation of toxic oligomers and boosting oxidative stress, respectively [8, 9]. Although the most considerable effect on AD development is attributed to Aβ_1-40_ and Aβ_1-42_ species, there is a huge diversity of Aβ peptides in vivo, especially considering the N-truncated forms, such as Aβ_p3–x_, Aβ_4–x_, Aβ_5–x_, Aβ_p11–x_, and Aβ_11–x_, with a potentially diverse role in the induction of oxidative stress [10–12]. Their amount, especially Aβ_4-x_, is at least as large as that of Aβ_1-x_ [13, 14]. In addition, the content of particular Aβ peptides differs between healthy people and AD patients [15], which could be employed in AD diagnosis. Recent studies also showed that brain enzymes such as neprilysin could further degrade Aβ peptides, resulting in short soluble forms [16]. What is noteworthy, the short length and similarity of amino acid sequences impede the detection of those peptides by standard methods, such as mass spectrometry and antibody-based assays. Thus, short-length Aβ peptides are perfect candidates for testing novel detection methods to discriminate structural analogs.

Recently, chemical tongue methods were proposed as a promising strategy for the detection of different forms of Aβ_1-40_ and Aβ_1-42_ aggregates [17–19]. However, the recognition of specific peptide sequences still poses a challenge. Our research group for the first time showed that QDs-assisted EEM fluorescence spectroscopy can be applied to recognize amino acids and several di- and tripeptides [6]. Then, we used a voltammetric chemical tongue to identify peptides within the group of Pro-Gly, carnosine, glutathione, Aβ_4-16_, and the α-factor N-terminus [20]. In our last paper, we showed that voltammetric tongue approach enabled recognition of sequence of Aβ_5-9_ derivatives, which presents the same Cu(II)-His2 coordination mode, but with modified residues at first and third positions [21].

Herein, we aimed to discriminate short Aβ peptides with high sequence resemblance. We used EEM fluorescence spectroscopy of thiomalic acid capped-CdTe quantum dots [22–24] as a cross-reactive receptor to discriminate various Aβ peptide sequences based on various interactions between the nanocrystals and the peptides.

## Results and discussion

As model compounds for testing discrimination ability of quantum dots-based “chemical tongue,” we selected seven amyloid β derivatives representing the most commonly studied models of the N-terminal sequences of Aβ peptides (Table 1, all experimental details are provided in Electronic Supplementary Information, ESI).

**Table 1:** Short-length Aβ peptides (free N-terminal amines and amidated C-termini)—amino acids sequences and basic properties

Abbrev	Aβ peptide sequence	pI	The charge at pH 7.4
Aβ_1-16_	DAEFRHDSGYEVHHQK-NH_2_	6.36	− 1
Aβ_4-16_	FRHDSGYEVHHQK-NH_2_	9.59	+ 1
Aβ_4-9_	FRHDSG-NH_2_	10.90	+ 1
Aβ_5-16_	RHDSGYEVHHQK-NH_2_	9.44	+ 1
Aβ_5-12_	RHDSGYEV-NH_2_	7.50	0
Aβ_5-9_	RHDSG-NH_2_	10.73	+ 1
Aβ_12-16_	VHHQK-NH_2_	13.91	+ 2

**Fig. 1:**
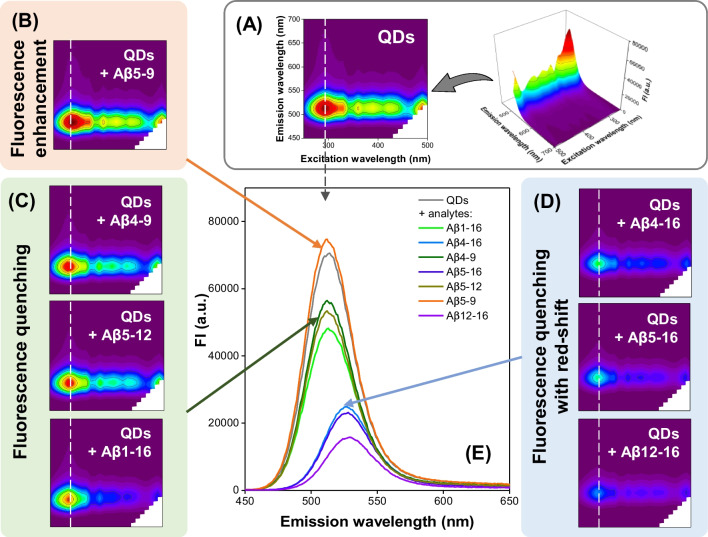
The influence of Aβ peptides on the fluorescence spectra of QDs. **A** EEM spectrum of QDs. **B**–**D** EEM spectra of QDs in the presence of 100 µM Aβ peptides showing their varied impact on QD fluorescence signal. **E** Fluorescence emission spectra under optimal excitation conditions (λ_ex_ = 290 nm). The spectra in **E** were extracted from respective EEMs in **A**–**D**

Their truncation at N- and C-termini resulted in a different number of acidic/basic residues and, in consequence, diverse peptide charges at pH 7.4. By applying QDs capping agent of anionic character, we assumed a possible varied affinity towards the nanomaterial’s surface [25]. The EEM spectra of QDs with and without Aβ peptides (details on their acquisition in Supplementary Information) are given in Fig. 1A–D. To more clearly illustrate Aβ-induced changes in QD’s fluorescence response, we extracted the fluorescence spectra acquired at the maximum excitation from EEM of each type (Fig. 1E).

Three different effects were observed in the EEMs of QDs influenced by their interaction with analytes at 100 µM: slight enhancement of the fluorescence signal at emission maximum ca 290 nm/510 nm (λ_ex_/λ_em_) in the case of Aβ_5-9_ (Fig. 1B); fluorescence quenching without any spectral shifts for Aβ_4-9_, Aβ_5-12_, and Aβ_1-16_ (Fig. 1C); or fluorescence quenching with red-shift of emission maximum in case of Aβ_4-16_, Aβ_5-16_, and Aβ_12-16_ (Fig. 1D). Moreover, the degree of the fluorescence quenching differed depending on the Aβ peptide sequence, in order Aβ_4-9_ < Aβ_5-12_ < Aβ_1-16_ < Aβ_4-16_ < Aβ_5-16_ < Aβ_12-16_ (Fig. 1E). Such varied EEM response of the applied QDs to the Aβ species proves their cross-selectivity and can be utilized as characteristic fingerprints in the chemical tongue approach.

**Fig. 2:**
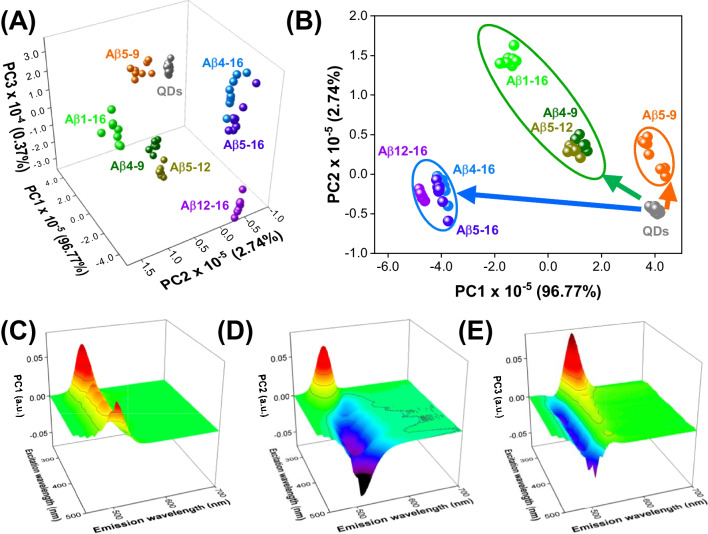
PCA model for the recognition of Aβ peptides by QDs-based chemical tongue. **A** Complete discrimination of Aβ peptides at 100 µM on the 3D score plot. **B** 2D score plot showing the grouping of various Aβ species according to their influence on EEMs of QDs. **C**–**E** Loading plots corresponding to PC1, PC2, and PC3, respectively

EEM fluorescent data of QDs in the presence of 100 µM Aβ peptides were unfolded and processed by principal component analysis (PCA) to implement the chemical tongue approach. PCA detects samples’ (dis)similarities by converting the original variables into principal components (PCs), capturing the maximum system variance [26]. PCA score plots (Fig. 2A, B) were used to discern the analytes, while the contribution of the original variables to the following PCs was observed on loadings plots (Fig. 2C–E; data analysis details in Supplementary Information). First of all, we achieved satisfactory discrimination of all investigated targets as the objects representing each Aβ peptide form well-separated clusters against three PCs included in the model (Fig. 2A). It must be underlined that the samples are grouped in PC1-PC2 space itself depending on the type of changes induced in the EEMs (compare Figs. 1 and 2B). The cluster of Aβ_5-9_, which slightly enhances the fluorescence of QDs, is placed close to the pure QDs cluster. The peptides that quench the fluorescence of QDs with a spectral shift (Aβ_4-16_, Aβ_5-16_, Aβ_12-16_) are characterized by negative PC1 and PC2, while compounds that only quench the fluorescence are grouped in near PC1 ≈ 0, having positive PC2 values. It is worth emphasizing that each PC provided complementary information for the analytes discrimination. PC3 is necessary to distinguish Aβ_4-16_ and Aβ_5-16_, as well as Aβ_4-9_ and Aβ_5-12_ (Fig. 2A), which slightly overlap when considering only PC1-PC2 space (Fig. 2B).

The loadings on PC1 reveal that it describes enhancing or quenching properties of Aβ peptides (Fig. 2C). Analytes that quench the fluorescence to the greatest extent (Aβ_1-16_, Aβ_5-16_, Aβ_4-16_, Aβ_12-16_) are characterized by negative PC1, while for remaining samples (Aβ_5-9_, Aβ_4-9_, Aβ_5-12_) it is positive (Fig. 2A). In turn, PC2 describes the differences in the obtained EEMs over λ_ex_ ∈ (250, 300 nm), λ_em_ ∈ (490, 520 nm), and λ_ex_ $\in$ (360, 500 nm), λ_em_ $\in$ (500, 550 nm), which allow distinguishing Aβ_5-12_, Aβ_4-9_, Aβ_5-9_, and Aβ_1-16_ (PC2 > 0) from the samples of Aβ_4-16_, Aβ_5-16_, and Aβ_12-16_ (PC2 < 0). PC3 contributes to the discrimination of Aβ_5-16_, Aβ_4-16_, Aβ_1-16_, Aβ_5-9_ with positive values on PC3, and Aβ_12-16_, Aβ_4-9_, Aβ_5-12_ exhibiting PC3 < 0 (Fig. 2A). The most influential spectral ranges for PC3 occur for λ_ex_ ∈ (250, 350 nm), λ_em_ ∈ (510, 560 nm), and λ_ex_ ∈ (280, 500 nm), λ_em_ ∈ (480, 510 nm), as presented in Fig. 2E. These findings confirm the necessity of using multispectral data to provide valuable discriminatory abilities of the developed QDs-based chemical tongue.

While investigating the mechanism behind the diversity of EEMs (Fig. 1), we first looked at His motifs in the peptide sequences (Table 1). It was reported that histidine-rich ligands are characterized by high electron transfer and metal binding abilities, which could highly alter the fluorescence signal of QDs [27]. Interestingly, all peptides causing the shifts of the fluorescence signal to longer wavelengths contain the *bis*-His motif (two adjacent histidine residues). On the other hand, the interaction of QDs with Aβ_1-16_, also containing a *bis*-His motif, did not induce spectral shifts in the resultant EEM. Analyzing the charge of the Aβ peptides at pH 7.4 (Table 1), it can be suspected that this difference results from the anionic character of the peptide caused by the presence of acidic amino acids (Glu and Asp residues) at the N-terminus of the Aβ_1-16_ sequence, which determines that it is the only negatively charged compound under conditions of our measurements. However, despite the negative charge of Aβ_1-16_, which should hinder the interaction with anionic QDs surface due to the electrostatic repulsion, this peptide quenches QDs fluorescence the most among all compounds that did not shift the emission maximum. It is also worth noting that the remaining Aβ peptides are mostly positively charged (only Aβ_5-12_ has a charge of 0), although they influence the fluorescent response of QDs in different ways (see Fig. 1). These observations imply that the observed effects might be a synergistic result of various types of QDs-Aβ interactions, including nanocrystals aggregation induced by electrostatic forces, and the possible affinity of Aβ peptides to the QDs surface, resulting from the presence of metal binding residues in Aβ sequence (as provided in Table 1).

To experimentally evaluate these hypotheses, we first examined susceptibility of QDs to peptide-induced aggregation with UV/vis spectroscopy (see Supplementary Information). For this purpose, we selected five Aβ peptides: two that quenched the fluorescence of QDs (Aβ_1-16_, Aβ_5-12_) and three that quenched the fluorescence of QDs with a red-shift of the emission maxima (Aβ_4-16_, Aβ_5-16_, Aβ_12-16_). No signs of aggregation were visible when 100 µM of both Aβ_1-16_ and Aβ_5-12_ were added to the solution of QDs (Figure S.1). In turn, the shift of absorbance spectra baseline, resulting from enhanced light scattering, was noted for Aβ_4-16_, Aβ_5-16_, and Aβ_12-16_. We can expect that the observed, Aβ peptide-triggered aggregation of QDs is a result of two phenomena: the overall affinity of a given peptide to the QD surface (which determines its surface adsorption) and the final QDs surface charge (responsible for the loss of colloidal stability).

**Fig. 3:**
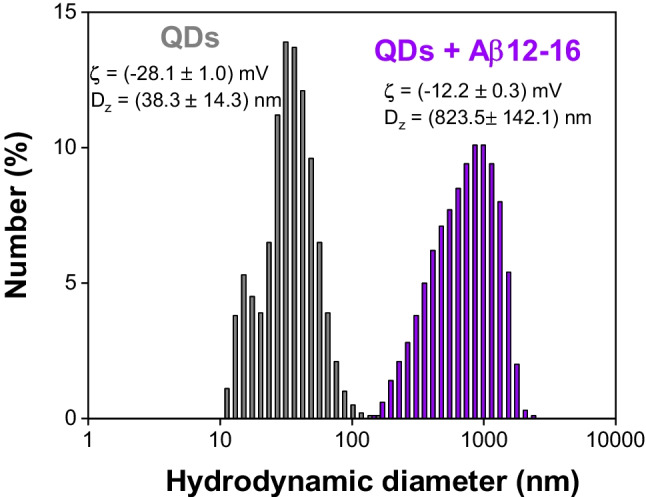
The hydrodynamic diameter and ξ-potential evolution caused by the aggregation of QDs in the presence of Aβ_12-16_

To further track these effects, we monitored the evolution of ζ-potential and the hydrodynamic diameter in the presence of selected Aβ peptides (see Fig. 3 and Supplementary Information, Table S.1). In all analyzed cases, the addition of the peptide resulted in attenuation of the initially strongly negative surface charge of QDs due to terminal carboxylate groups of the capping agent (Table S1). The peptide-induced QDs aggregation was also reflected in the increase of hydrodynamic diameters (Fig. 3). The highest ζ-potential shift was observed for the cationic peptides Aβ_4-16_, Aβ_5-16_, and Aβ_12-16_. Interestingly, the neutral peptide (Aβ_5-12_) and the anionic peptide (Aβ_1-16_) showed a similar minor capability for colloidal destabilization of QDs. This suggests that the adsorption of the Aβ_1-16_ peptide remains at an equivalent level to that of the Aβ_5-12_ due to the counteracting electrostatic effect under the given pH and ionic strength.

Since the applied QDs have a CdTe core, variability of the obtained EEM fluorescent response might also derive from a possible varied affinity of Aβ species towards the nanocrystal’s surface rich in Cd(II) ions [28], especially given that the bis-His motif enhances the stability of Cd(II) complexes [29]. Therefore, we performed spectrofluorimetric titration experiments with CdCl_2_ (see Supplementary Information). For these measurements, we chose the peptides containing a Tyr residue (Aβ_1-16_, Aβ_4-16_, Aβ_5-16_, and Aβ_5-12_) as the fluorescence spectra were collected at the characteristic Tyr excitation wavelength of 275 nm (similar methods were previously used in Cu(II)-Aβ coordination studies) [10, 30]. Although the obtained results should be considered as a preliminary description of the potential interaction between Aβ peptides and Cd(II), we noticed significant differences in responses of Aβ peptides to Cd(II). The effect of subsequent additions of Cd(II) ions to the Aβ_5-12_ sample was negligible (Figure S.2A), suggesting a very weak, if any, interaction between Cd(II) and Aβ_5-12_ (the peptide without the *bis*-His motif). In contrast, we observed an increase in the fluorescence signal in the course of the titration of Aβ_1-16_, Aβ_4-16_, and Aβ_5-16_, which suggests the binding of Cd(II) ions to the Aβ peptides possessing the *bis*-His motif (Figure S.2B-E). Thus, we showed that the changes in the EEM spectra (Fig. 1), which are indispensable for recognizing short-length Aβ peptides (Fig. 2), are likely driven by multiple QDs-peptide interactions. We do not exclude other mechanisms that could affect the peptides’ discrimination.

The following experiment aimed to evaluate the potential of our method in quantitative analysis of Aβ peptides. As an example, PCA score plot obtained for Aβ_4-16_ samples at various concentration levels revealed that perfect discrimination in PC1-PC2 space was obtained for concentrations: 100 μM, 10 μM, 1 μM, and 100 nM (see Supplementary Information, Figure S.3A). Hierarchical cluster analysis (HCA) confirmed the same sensitivity at micromolar range (Figure S.3B). The response of QDs-based chemical tongue was similar to pure QDs for Aβ_4-16_ at nanomolar level. To estimate the limit of detection more precisely, independent PCA for nano- and pico-molar concentration range was performed (Figure S.3C) and PC1 score served as an estimate of overall response of the chemical tongue. For the series of concentrations, two-tailed *t*-tests detected significant differences in PC1 score when comparing with pure QDs (Figure S.3D). In this way, limit of detection was found at the level of 1 nM of Aβ_4-16_ (1 pmol/mL; 1.6 ng/mL).

Finally, to prove the potential of the presented method in the analysis of peptide mixtures, 48 samples containing Aβ_1-16_, Aβ_4-16_, and Aβ_5-9_ were prepared (details in Supplementary Information, Figure S.4). Their choice was dictated by their various influences on the QDs-based chemical tongue response: fluorescence quenching (Aβ_1-16_), quenching with red-shift (Aβ_4-16_), or fluorescence enhancement (Aβ_5-9_). Moreover, Aβ_1-16_ and Aβ_4-16_ are the models of the most common Aβ forms already detected in biological samples. Detection of the individual Aβ peptide at 100 μM in binary and ternary mixtures was performed by QDs-based chemical tongue using partial least squares-discriminant analysis (PLS-DA). Perfect, 100% accuracy was achieved for the two studied peptides (Aβ_4-16_ and Aβ_4-16_), while for the third one (Aβ_5-9_) it was slightly lower (97.9%, Figure S.4). It proves that our method is highly promising for the short-length peptide determination in more complex systems, which will be further explored in our future projects.

## Conclusions

The recognition of small peptides provides a significant challenge for the standard methods dedicated mostly to proteins and long peptides. Therefore, their presence in biological samples could be overlooked or underestimated as they are often easily washed out from chromatographic columns with other low molecular weight molecules, wrongly assigned as the fragments of proteins or longer peptides during analysis of mass spectrometry results, or not recognized by antibodies [15, 31]. In light of the above, the excellent recognition of small and structurally related Aβ peptides presented by our method, employing QDs as receptors, EEM as the detection method, and machine learning to analyze the data, is highly promising for studies on physiologically important small peptides.

Aβ peptides are known as important biomarkers for Alzheimer’s disease. However, despite the huge variety of their analogs, the ratio of long forms Aβ_1-42_:Aβ_1-40_ is exclusively used in AD diagnostics. The previous works on the chemical tongue towards Aβ peptides were also focused on long forms applying polyelectrolytes and organic dyes [17], dendrimers [19], gold and silver nanoparticles [32, 33] as receptors, but not QDs. On the other hand, sensing by means of fluorescent nanomaterials is mainly focused on interpreting scalar signals (fluorescence intensity at fixed excitation and emission wavelengths or fluorescence shift), while various mechanisms involved in such processes can provide effective and significant enrichment of analytical information. In this work, we showed that combining these two features, which are the application of QDs and proper decoding of their multispectral response (EEM spectrum), leads to high discriminatory power towards recognizing Aβ derivatives characterized by high structural similarity. Furthermore, this system is potentially easy to modify, for example, by changing the QD’s ligands to provide additional, more specific interactions, lowering the detection limit, and as such be a potentially attractive alternative to analyze clinical samples, which is the aim of our future projects. Moreover, the presented results are important not only from the perspective of the possible development of new detection methods for short-length peptide research but also can suggest that reinterpretation of existing nano-detection systems in terms of enhancing their multidimensional information with further data exploration can be beneficial for improved bioanalytical performance and better understanding of mechanisms at the QD-analyte interface.

### Supplementary Information

Below is the link to the electronic supplementary material.

Supplementary file1 (PDF 799 KB)

## Data Availability

Data available on request from the authors.
