# Supplementary material for: Quantum dots-based “chemical tongue” for the discrimination of short-length Aβ peptides
Source: Mikrochim Acta. 2024 Jan 15;191(2):95. doi: 10.1007/s00604-023-06115-0 (PMC10789672; doi:10.1007/s00604-023-06115-0)
Supplement: Supplementary file 1 — Supplementary file1 (PDF 799 KB) [file 604_2023_6115_MOESM1_ESM.pdf]

Electronic Supplementary Information for Quantum dots-based 'chemical tongue' for the discrimination of short-length Aβ peptides

Klaudia Głowacz [a], Marcin Drozd [a,b], Weronika Tokarska [a], Nina E. Wezynfeld [a], Patrycja Ciosek-Skibińska [a]**

[a] Chair of Medical Biotechnology, Faculty of Chemistry, Warsaw University of Technology, Noakowskiego 3, 00-664 Warsaw, Poland

[b] Centre for Advanced Materials and Technologies CEZAMAT, Poleczki 19, 02-822, Warsaw, Poland

*Correspondance: patrycja.ciosek@pw.edu.pl (PCS), klaudia.glowacz.dokt@pw.edu.pl (KG)

Table of content

1. Materials and methods 3

1.1 Reagents and materials 3

1.2 Aβ peptides discrimination 3

1.3 The acquisition of EEM spectra 4

1.4 Data analysis 4

1.5 UV-vis spectroscopy 5

1.6 ζ-potential and dynamic light scattering measurements 5

1.7 Spectrofluorometric titration 5

2. Aβ peptides-induced agglomeration of QDs-TMA 7

3. Studies on the potential interactions between Aβ peptides and Cd(II) ions 9

4. Quantitative analysis of $Aβ_{4-16}$ ……………………………..…………………..………10

5. Analysis of mixtures of Aβ peptides ……………………………………………...…...11

1. Materials and methods

1.1 Reagents and materials

Thiomalic acid-capped quantum dots with CdTe core (QDs), a diameter of 1.5 nm, and λ_{max} = 510 nm were obtained from PlasmaChem GmbH (Berlin, Germany). The investigated amyloid β (Aβ) peptides were synthesized according to the Fmoc strategy[1] and purchased from the Institute of Biochemistry and Biophysics PAS (Warszawa, Poland). The concentration of the Aβ stock solutions were determined with UV-Vis spectroscopy, using an extinction coefficient related to Tyr ($\varepsilon_{276–296}$ = 1410 cm^{-1} M^{-1}) for $A\beta_{1-16}$, $A\beta_{4-16}$, $A\beta_{5-16}$, and $A\beta_{5-12}$ or by UV-Vis titrations of the peptide solution with Cu(II) for $A\beta_{4-9}$, $A\beta_{5-9}$, $A\beta_{12-16}$.[2] N-(2-hydroxyethyl)piperazine-N'-(2-ethane sulfonic acid) (HEPES) and cadmium chloride were obtained from Sigma-Merck (Poznań, Poland). Milli-Q water was used for the preparation of all aqueous solutions. All reagents were used as received.

1 W. C. Chan and P. D. White, in *InFmoc Solid Phase Peptide Synthesis, A Practical Approach*, eds. W. C. Chan and P. D. White, Oxford University Press, New York, NY, 2000, pp. 41–76.

2 N. E. Wezynfeld, A. Tobolska, M. Mital, U. E. Wawrzyniak, M. Z. Wiloch, D. Płonka, K. Bossak-Ahmad, W. Wróblewski and W. Bal, *Inorg. Chem.*, 2020, **59**, 14000–14011.

1.2 Aβ peptides discrimination

The samples for Aβ peptides discrimination were prepared in UV-Star® 96-well plates (Greiner Bio-One GmbH, Kremsmünster, Austria). For each of the investigated analytes ($A\beta_{1-16}$, $A\beta_{4-16}$, $A\beta_{4-9}$, $A\beta_{5-16}$, $A\beta_{5-12}$, $A\beta_{5-9}$, $A\beta_{12-16}$), the solution of QDs in 50 mM HEPES buffer at pH 7.4 was first pipetted to each well. Next, 2 µL of aqueous solutions of Aβ peptides were added so that the final concentration of QDs and analyte in each sample type was 25 µg/mL and 100 µM, respectively. The volume of all samples was 200 µL. The control samples of QDs were prepared by adding 2 µL of deionized water instead of Aβ peptide solution. Each sample type was prepared

in 8 independent replications. The samples were subjected to excitation-emission matrix fluorescence spectroscopy measurements immediately after preparation.

1.3 The acquisition of EEM spectra

Excitation-emission matrices (EEMs) of QDs in the presence of Aβ peptides were recorded with a Synergy™ Neo 2 Hybrid Multi-Mode Microplate Reader fluorescence spectrometer (BioTek Instruments, Inc., Winooski, VT, USA). For this purpose, a hand-written protocol was used that relies on recording consecutive emission spectra at increasing excitation wavelengths. First, samples prepared in a 96-well plate were mixed for 1 min. Then, the emission spectra of each sample were recorded by changing the excitation wavelengths from 250 nm to 500 nm, $\Delta\lambda_{ex}$ = 10 nm. The range of the recorded emission spectra depended on the excitation wavelength at which the spectrum was acquired to avoid Rayleigh signals. Thus, for $\lambda_{ex} \in$ (250 nm, 430 nm), the emission was recorded in the range of 450 nm -700 nm, whereas for $\lambda_{ex} \in$ (440 nm, 500 nm), fluorescence spectra were acquired at $\lambda_{em} \in (\lambda_{ex} + 20$ nm, 700 nm). All emission spectra were registered with a constant $\Delta\lambda_{em}$ = 1 nm. All experiments were performed at room temperature.

1.4 Data analysis

The conducted EEM fluorescence spectroscopy measurements resulted in a collection of 26 emission spectra per sample. The obtained fluorescence spectra describing each sample were arranged in the excitation-emission matrices, consisting of all fluorescence intensities obtained for respective excitation and emission wavelengths (26×251 EEM characterized each sample). Unfolded Principal Component Analysis (unfolded PCA), unfolded Hierarchical Cluster Analysis (HCA), and unfolded Partial Least Squares-Discriminant Analysis (unfolded PLS-DA) were used for Aβ peptides qualitative or quantitative analysis. Therefore, before every model establishment, data vectors corresponding to each emission spectra obtained at the following excitation wavelengths were arranged side by side. Each sample was described by a data vector of 1×6246 (missing data resulting from the measurement procedure were omitted). Thus, the data matrix for

PCA qualitative analysis was 72 x 6246, for PCA exploring the potential of quantitative analysis was 60 x 6246, for PLS-DA was 48 x 6246, and for HCA was 42 x 6246 (samples × all fluorescence intensity values at respective $\lambda_{ex}/\lambda_{em}$). Mean-centering was used as a preprocessing step. Chemometric analysis was performed in Solo 9.1 (Eigenvector Research Inc., Manson, USA). Figures were created in Origin (OriginLab Corporation, Northhampton, MA, USA) software.

1.5 UV-vis spectroscopy

UV-vis measurements were carried out to initially assess the occurrence of Aβ peptides-induced QDs agglomeration. UV-vis spectra of QDs, Aβ peptides, as well as mixtures of QDs and Aβ peptides, were recorded at room temperature on a Cary 60 spectrophotometer (Agilent, Santa Clara, CA, USA) over a spectral range of 200-1000 nm using a 1 cm-path quartz cuvette (Hellma GmbH & Co, Müllheim, Germany). Samples were prepared to maintain the conditions used during the collection of EEMs (see subsection 1.2).

1.6 ζ-potential and dynamic light scattering measurements

ζ-potential and dynamic light scattering (DLS) measurements were conducted to further study the impact of QDs interactions with an individual Aβ peptide on the evolution of QD' surface charge and the degree of interaction-induced aggregation, respectively. Measured samples were prepared to simulate the conditions applied during EEMs acquisition (see subsection 1.2). Both experiments were performed by means of a Zetasizer Nano ZS (Malvern Panalytical Ltd., Malvern, UK) at 25 °C. The disposable polystyrene cuvettes and dip cell for ζ-potential measurements were used. The experiments were conducted at a detection angle of 173° using a He-Ne laser (power 4 mW, wavelength 632.8 nm) as a light source.

1.7 Spectrofluorometric titration

Spectrofluorimetric titrations of Aβ peptides with aqueous $CdCl_2$ solution were carried out using a FluoroMax®-3 spectrofluorometer (Horiba Jobin Yvon, Longjumeau Cedex, France) at

the excitation wavelength of 275 nm (slit widths for emission were 2 nm, whereas for excitation 3 nm) in quartz cuvettes of path length = 1 cm (Hellma GmbH & Co, Müllheim, Germany). The fluorescence intensity was registered for the emission range of 290-400 nm. Salt solutions containing the appropriate level of Cd(II) ions were added to the respective Aβ peptide solution in 50 mM HEPES at pH 7.4, changing the Cd(II): peptide molar ratio from 0.2 to 5.0.

2. Aβ peptides-induced aggregation of QDs

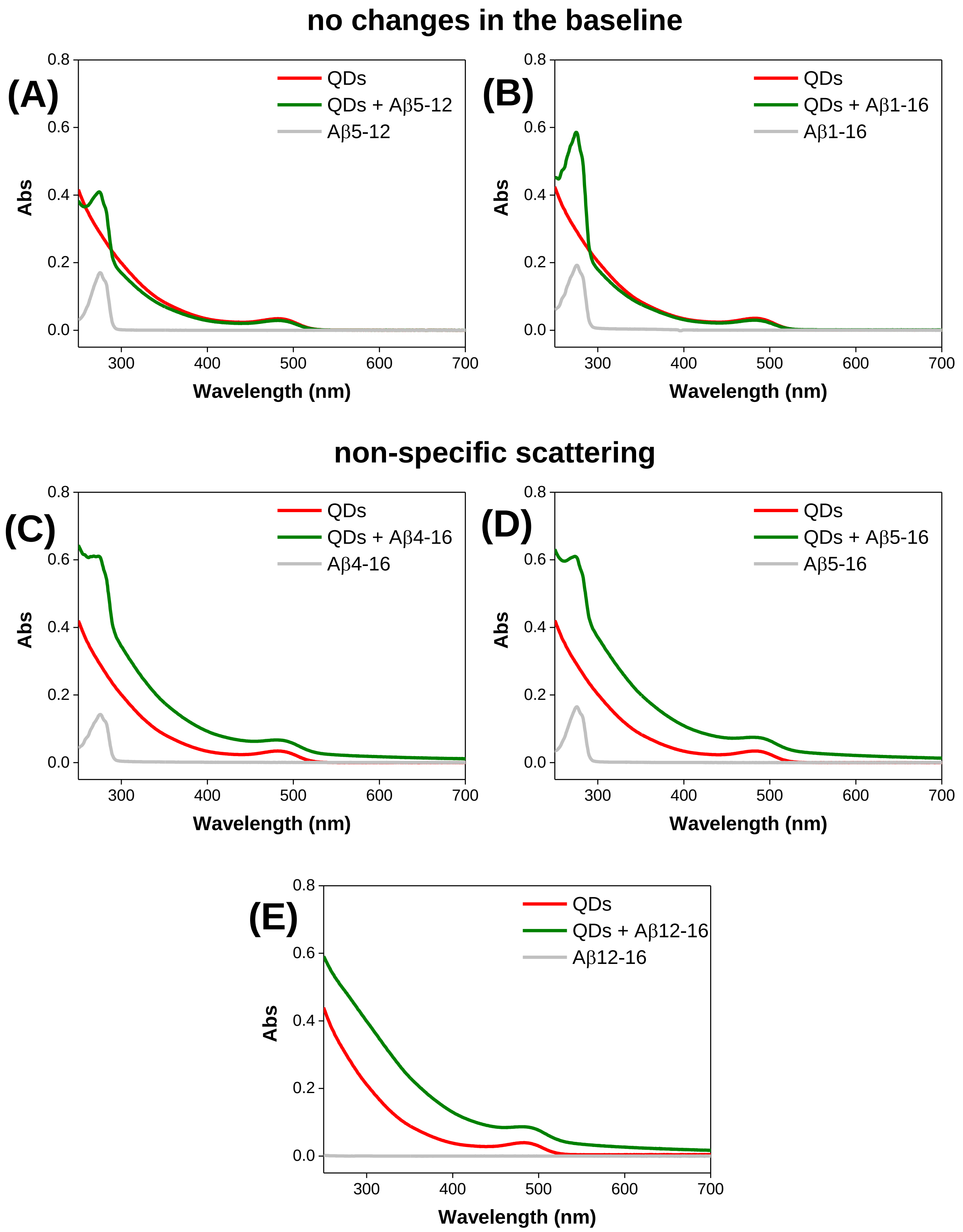

Figure S. 1. The changes in the baseline of absorption spectra showing (A,B) no agglomeration signs or (C-E) non-specific scattering under conditions of analytes detection (25 µg/mL QDs, 100 µM Aβ, 50 mM HEPES, pH 7.4): (A) $A\beta_{5\text{-}12}$, (B) $A\beta_{1\text{-}16}$, (C) $A\beta_{4\text{-}16}$, (D), $A\beta_{5\text{-}16}$ (E) $A\beta_{12\text{-}16}$. For clarity, only spectral range of 250-700 nm was presented.

Table S. 1. The mean values (± SD, n = 5) of ζ-potential and hydrodynamic diameter of QDs without and in the presence of Aβ peptides (25 µg/mL QDs, 100 µM Aβ, 50 mM HEPES, pH 7.4).

Sample type	ζ potential (mV)	Hydrodynamic diameter (nm)
QDs	-28.1 ± 1.0	38.3 ± 14.3
QDs + $A\beta_{1-16}$	-23.0 ± 0.8	86.0 ± 14.3
QDs + $A\beta_{5-12}$	-22.7 ± 1.3	73.6 ± 24.9
QDs + $A\beta_{4-16}$	-14.1 ± 0.6	$1.03 \cdot 10^3 \pm 0.12 \cdot 10^3$
QDs + $A\beta_{5-16}$	-14.0 ± 0.3	276 ± 89
QDs + $A\beta_{12-16}$	-12.2 ± 0.3	824 ± 142

3. Studies on the potential interactions between Aβ peptides and Cd(II) ions

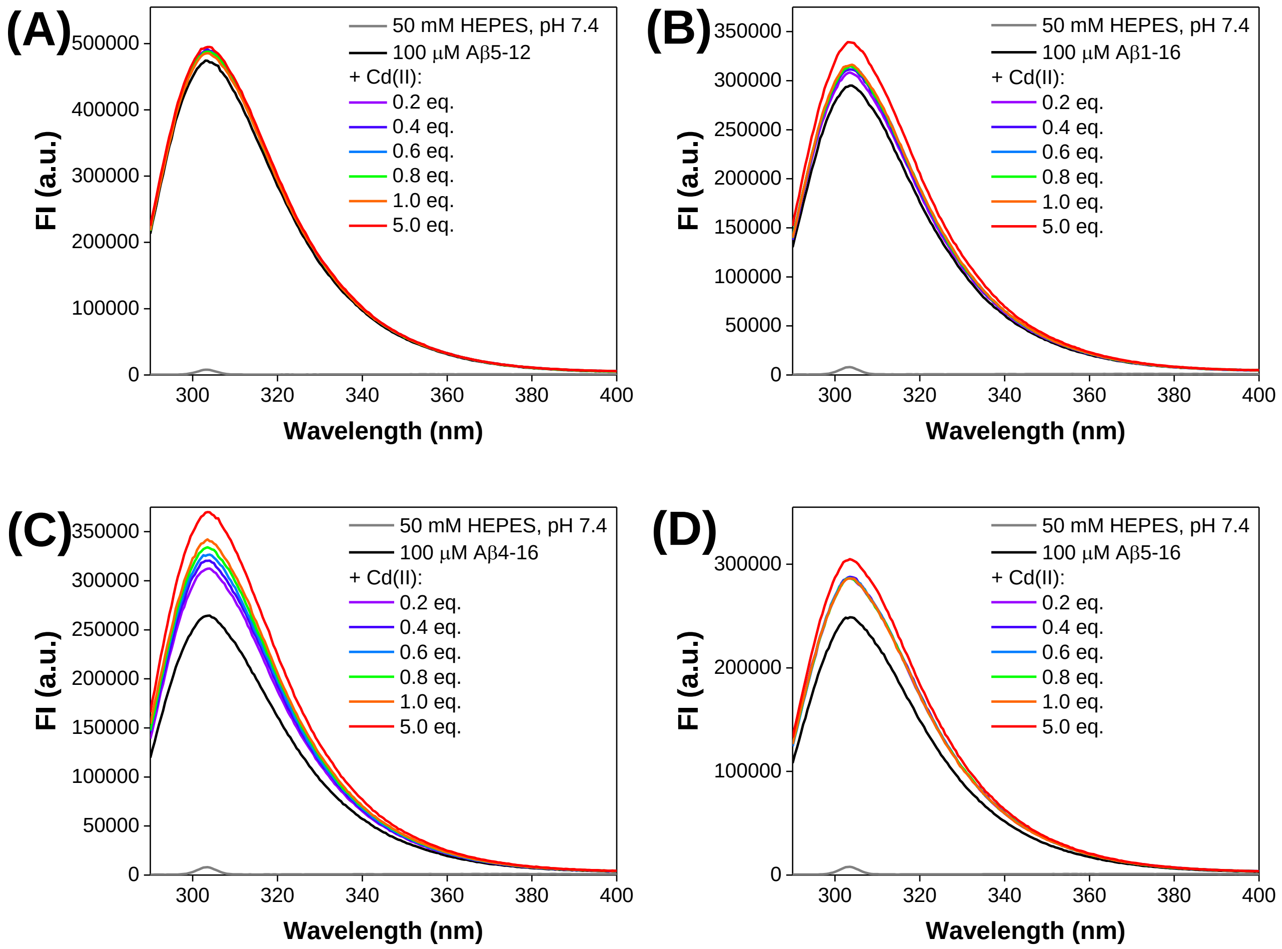

Figure S. 2. Spectrofluorometric titration of Aβ peptides with $CdCl_2$ (100 µM Aβ, 50 mM HEPES, pH 7.4): (A) $A\beta_{5-12}$, (B) $A\beta_{1-16}$, (C) $A\beta_{4-16}$, (D) $A\beta_{5-16}$. The emission spectra were acquired for λ_{ex} = 275 nm to observe eventual evolution of tyrosine residue fluorescence.

4. Quantitative analysis of Aβ4-16

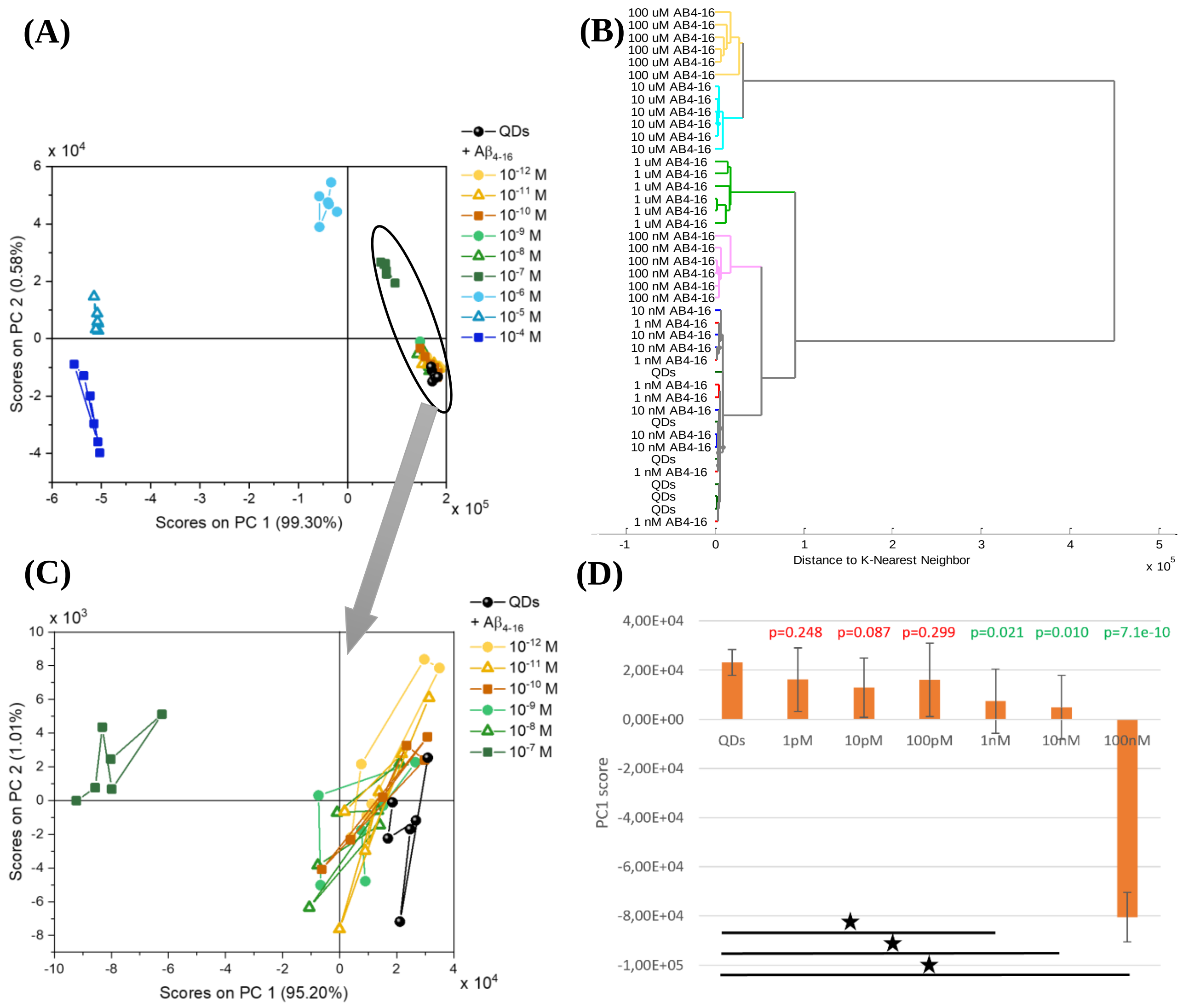

Figure S. 3. Quantitative analysis of Aβ4-16 performed by QDs-based chemical tongue: (A) PCA score plot showing discrimination of samples at various concentration levels; (B) Hierarchical Cluster Analysis of Aβ4-16 samples in concentration range 0-100 μM (Euclidean distance for mean-centered data was applied); (C) PCA score plot showing discrimination of Aβ4-16 samples at nano- and pico-molar concentration range; (D) QDs-based chemical tongue response towards Aβ4-16 at nano- and pico-molar concentration level, shown as PC1 score (extracted from (C), mean±SD, n=6). The results of two-tailed t-tests as p-values show significant (green, $p < \alpha$, $\alpha=0.05$) and insignificant (red, $p \geq \alpha$, $\alpha=0.05$) differences noticed for various concentration levels when comparing with pure QDs. The limit of detection was found at 1 nM of Aβ4-16.

5. Analysis of mixtures of Aβ peptides

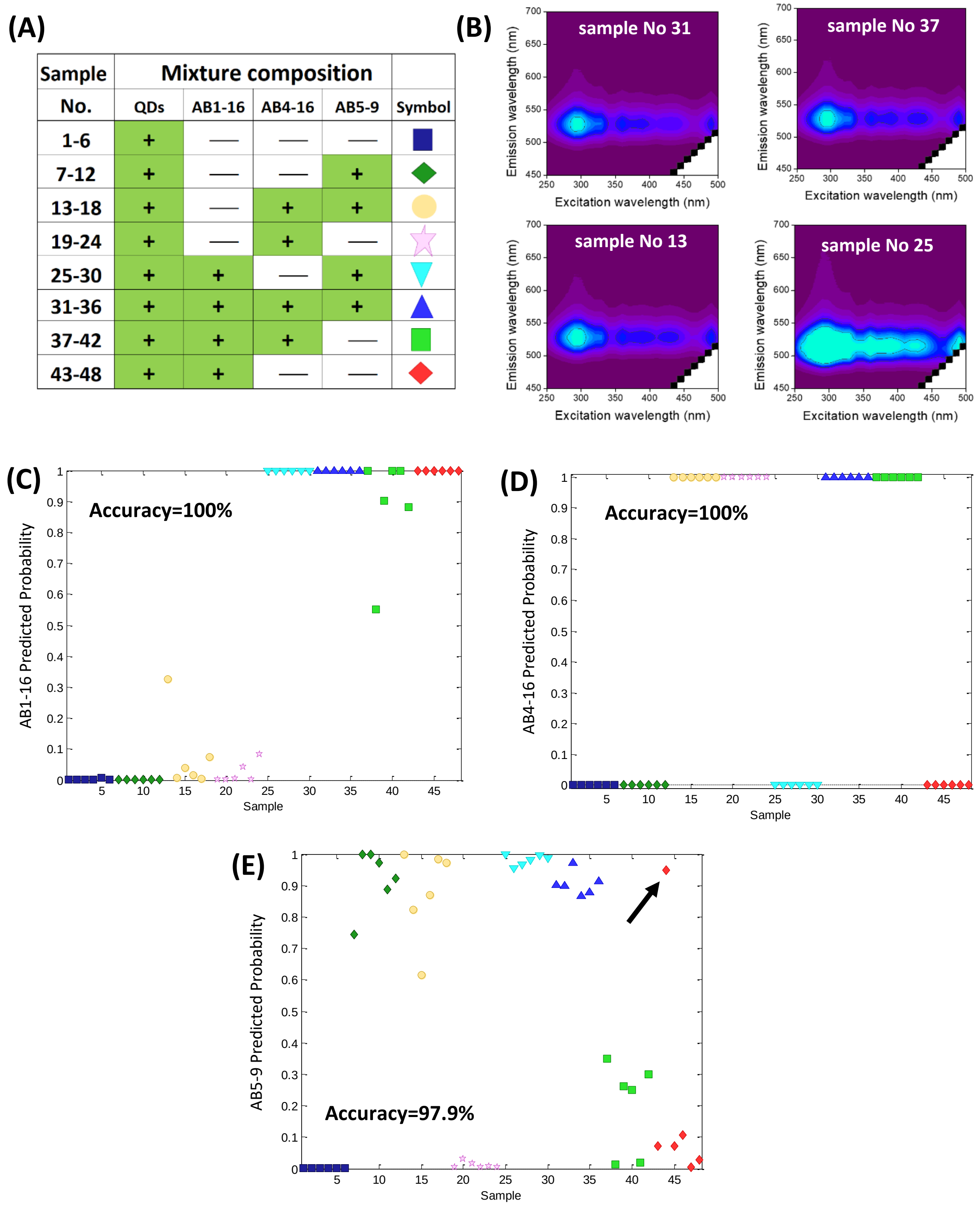

Figure S. 4. The detection of the individual Aβ peptide (25 µg/mL QDs, 100 µM Aβ, 50 mM HEPES, pH 7.4) in binary and ternary mixtures performed by QDs-based chemical tongue using Partial Least Squares-Discriminant Analysis: (A) composition of 48 studied samples; (B) exemplary EEMs; (C),(D),(E) predicted probability of the presence of $A\beta_{1\text{-}16}$, $A\beta_{4\text{-}16}$, $A\beta_{5\text{-}9}$ in the following samples, respectively. In the case of $A\beta_{1\text{-}16}$ and $A\beta_{4\text{-}16}$ detection was successful for all samples, whereas in the case of $A\beta_{5\text{-}9}$ one false positive was observed (44th sample marked with an arrow on (E)).
